# Sensitive and selective detection of Mucin1 in pancreatic cancer using hybridization chain reaction with the assistance of Fe_3_O_4_@polydopamine nanocomposites

**DOI:** 10.1186/s12951-022-01289-w

**Published:** 2022-02-23

**Authors:** Qing Dong, Xiuna Jia, Yuling Wang, Hao Wang, Qiong Liu, Dan Li, Jin Wang, Erkang Wang

**Affiliations:** 1 College of Chemistry, Jilin University, Changchun, 130012 Jilin People’s Republic of China; 2 State Key Laboratory of Electroanalytical Chemistry, Changchun Institute of Applied Chemistry, Chinese Academy of Sciences, Changchun, 130022 Jilin People’s Republic of China; 3 ARC Centre of Excellence for Nanoscale BioPhotonics, Department of Molecular Sciences, Macquarie University, Sydney, 2109 Australia; 4 Department of Chemistry and Physics, State University of New York at Stony Brook, Stony Brook, NY 11794-3400 USA

**Keywords:** Pancreatic cancer detection, Hybridization chain reaction, Imaging, Dopamine coated magnetic composites

## Abstract

Pancreatic cancer is characterized as the worst for diagnosis lacking symptoms at the early stage, which results in a low overall survival rate. The frequently used techniques for pancreatic cancer diagnosis rely on imaging and biopsy, which have limitations in requiring experienced personnel to operate the expensive instruments and analyze the results. Therefore, there is a high demand to develop alternative tools or methods to detect pancreatic cancer. Herein, we propose a new strategy to enhance the detection sensitivity of pancreatic cancer cells both in biofluids and on tissues by combining the unique property of dopamine coated Fe_3_O_4_ nanoparticles (Fe_3_O_4_@DOP NPs) to specifically quench and separate free 6-carboxyfluorescein (FAM) labeled DNA (H_1_-FAM/H_2_-FAM), and the key feature of hybridization chain reaction (HCR) amplification. We have determined the limit of detection (LOD) to be 21 ~ 41 cells/mL for three different pancreatic cancer cell lines. It was also discovered that the fluorescence intensity of pancreatic cancer cells was significantly higher than that of HPDE-C7 and HepG-2 cells (control cell lines), which express lower MUC1 protein. Moreover, the HCR amplification system was used to identify the cancer cells on pancreatic tissue, which indicated the versatility of our strategy in clinical application. Therefore, the presented detection strategy shows good sensitivity, specificity and has great potential for the diagnosis of pancreatic cancer.

**Graphical Abstract:**

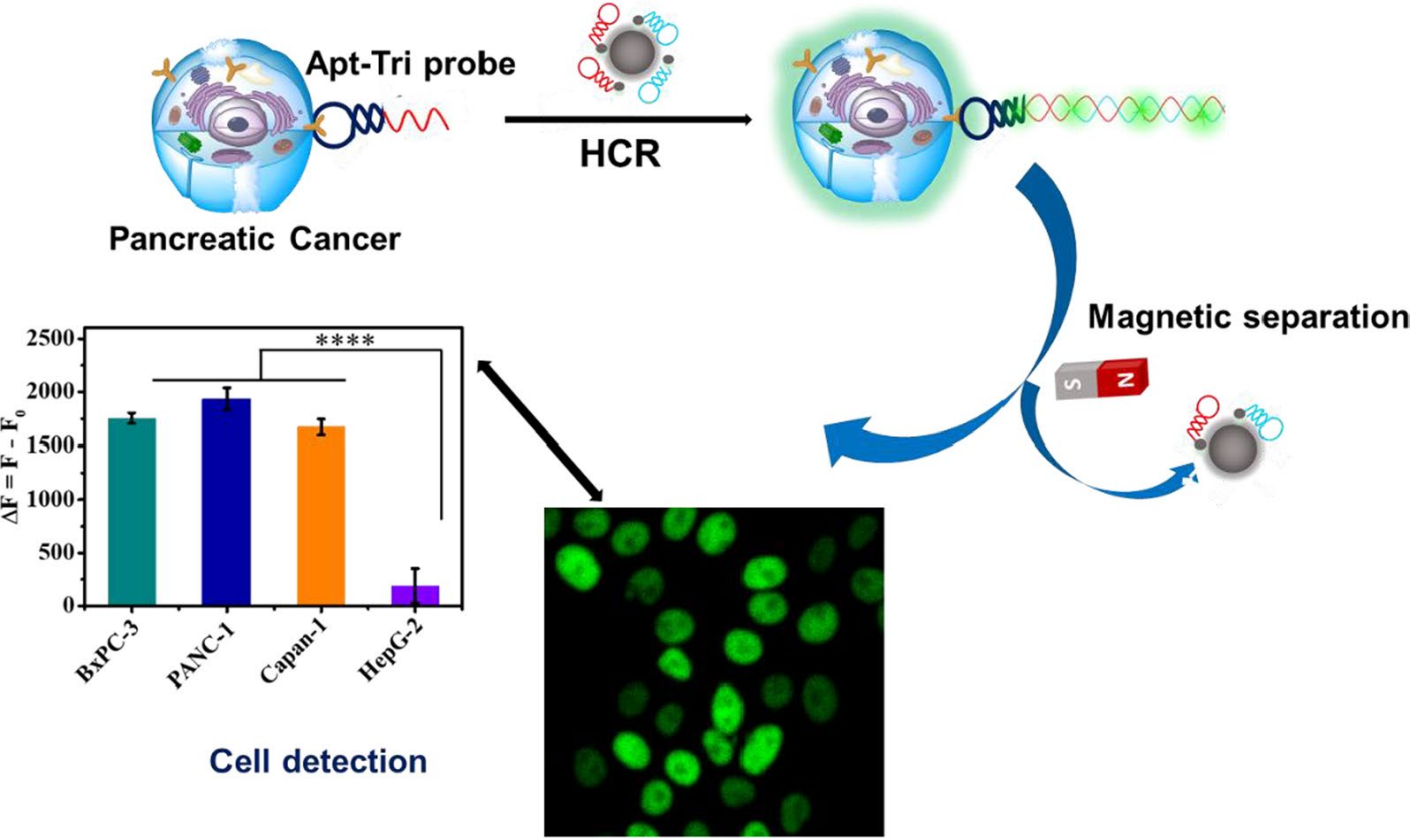

**Supplementary Information:**

The online version contains supplementary material available at 10.1186/s12951-022-01289-w.

## Introduction

Pancreatic cancer with a five-year survival rate of < 9%, is identified as one of the most lethal cancers in the world [1]. Due to the lack of specific clinical symptoms at its early stage, about 80% of patients have lost the opportunity for surgical resection [2]. Although not ideally sensitive and specific as expected, the biomarker, serum carbohydrate antigen 19-9 (CA19-9), has been used as a criteria in the diagnosis of pancreatic cancer [3]. However, the use of this biomarker can potentially bring false positive results. Therefore, alternative biomarkers are demanded to assure the accuracy. In addition, the current analytical techniques and imaging methods, such as Raman imaging [4], photoacoustic imaging [5] and magnetic resonance imaging [6], have been used in cancer diagnosis. These methods bring the advantages of multiple capacity, high spatial resolution and deep tissue penetration in soft tissue detection of the targets. While, these technologies have some deficiencies exactly, such as time-consuming, expensive and requiring trained professionals to use the instruments and analyze the results. Therefore, it is essential to design easy operating strategies with high sensitivity and specificity for the diagnosis of pancreatic cancer both in the biofluids and medical imaging. With the rapid development of nanobiotechnology, the detection strategy possessing the advantages of high sensitivity, simplicity, flexibility and low cost will be more adaptable.

Mucin1 (MUC1) is a transmembrane glycoprotein highly expressed both in malignant tumors and precancerous lesions [7]. MUC1 expresses in several human epithelial malignancies including pancreatic, gastric, colorectal, breast, endometrial, lung, bladder, and renal cell cancers [8]. Over-expression of MUC1 reduces the adhesion of cancer cells with the outer matrix, so that the cancer cells easily detach from the original site and lead to the metastasis [9, 10]. As a cancer associated protein, MUC1 has also been used as a biomarker for the diagnosis of pancreatic cancer [11–13]. Therefore, developing a method that can profile or quantify the expression of MUC1 is promising in diagnosis or monitoring the treatment of pancreatic cancer. Aptamer (Apt) is a single stranded DNA or RNA that can recognize and bind to their target molecules selected through the systematic evolution of ligands by exponential enrichment (SELEX) technology [14, 15]. It has been reported that the MUC1 Apt has high affinity to MUC1 protein with the disassociation constant (*k*_*d*_) of 1.35 × 10^–8^ M [16], which has been applied in the diagnosis of pancreatic cancer [17–19].

The hybrid chain reaction (HCR) is an isothermal, enzyme-free DNA amplification method that can be performed at room temperature [20–22]. No enzymes are required in the HCR process, which is cost-effective and can streamline the detection procedure. Zhao et al. used HCR amplification to determine K-RAS gene mutation in pancreatic cancer, and the limit of detection (LOD) reached as low as 15 pM [23]. HCR amplification was also used to accurately profile the exosomes of pancreatic cancer, and the results showed the LOD down to 2.2 × 10^3^ exosomes/mL [24]. These reports indicate that the HCR has the excellent performance in signal amplification and has been applied in the detection of cancer biomarkers.

Fe_3_O_4_ magnetic nanoparticles have the advantages of easy separation, low cost, recyclability, and have been used in the detection of cancer cells [25, 26]. After being coated with dopamine (DOP), the Fe_3_O_4_@DOP NPs possess excellent adsorption properties for single stranded DNA (ssDNA) through π–π stacking [27]. Furthermore, taking into account of the nearly full spectrum absorption ability of poly-dopamine, the fluorescence of the fluorophores will be quenched through the fluorescence resonance energy transfer (FRET) effect. Compared with the generally used organic quenchers [28], such as black hole quencher (BHQ), which can only specifically quench the fluorescence of certain fluorophores, the low cost and facile to be synthesized Fe_3_O_4_@DOP NPs as the nanoquenchers are more versatile that can significantly quench different fluorescence molecules at the same time [29]. Thus, Fe_3_O_4_@DOP NPs have been demonstrated with the applications in the detection of ssDNA [30], ATP [31] and living cells [32]. However, the fluorescent sensor for the pancreatic cancer detection using Fe_3_O_4_@DOP NPs as a fluorescence quencher has not been reported.

In this study, we utilized the fluorescence quenching and easy separation abilities of Fe_3_O_4_@DOP NPs, and a locked MUC1 molecular probe: aptamer-trigger (Apt-Tri) for assisting signal contrast enhancement in the detection of MUC1 overexpressed pancreatic cancer cells. The well designed Apt-Tri with alleviated signal leaking including two segments: MUC1 aptamer (Apt) for the recognition of pancreatic cancer cells and the trigger (Tri) for initiating the HCR amplification. When pancreatic cancer cells are present in the PBS buffer, the MUC1 Apt in the probe will bind to the MUC1 protein through the specific recognition, and release the Tri for starting the HCR amplification. With the addition of Fe_3_O_4_@DOP NPs carrying quenched H_1_-FAM/H_2_-FAM, the exposed Tri will hybridize with H_1_-FAM/H_2_-FAM to form a long strand duplex, and the fluorescence signal around the target cells will be enhanced due to the release of the FAM molecules from the surface of Fe_3_O_4_@DOP NPs. Accordingly, the extra FAM labelled hairpin DNA and MUC1 probes absorbed on Fe_3_O_4_@DOP NPs will be separated by external magnet. As a result, the fluorescence intensity is proportional to the concentration of pancreatic cancer cells. The linear detection ranges of three pancreatic cancer cell lines are 50–10^5^ cells/mL, and the LOD is in the range of 21 ~ 41 cells/mL. Furthermore, this enzyme-free and highly sensitive fluorescent sensor is performed directly in the fluorescence imaging of pancreatic cancer cells on tissues. In short, the strategy using MUC1 probe, HCR amplification and Fe_3_O_4_@DOP NPs shows the potential applications for pancreatic cancer detections. The presented detection approach can open new avenues on aptamer based recognition of other pancreatic cancer biomarkers.

## Materials and methods

### Materials

All the DNA oligonucleotides were purchased from Sangon Biotech Co., Ltd. (Shanghai, China). The DNAs labelled with FAM or ROX were purified by high-performance liquid chromatography (HPLC) and the sequences were listed in Additional file 1: Table S1 in the Supporting Information. FeCl_3_·6H_2_O, polyethylene glycol (MW 2000), ethylene glycol and dopamine hydrochloride were supplied from Sigma-Aldrich (St. Louis, MO, USA). The human pancreatic cell lines (Capan-1, BxPC-3 and PANC-1), a normal human pancreatic cell line (HPDE-C7) as well as the hepatocellular carcinoma (HepG-2) cells were purchased from American Type Culture Collection (ATCC, Rockville, MD, USA). Fetal bovine serum (FBS, Hyclone, USA), the culture reagents and penicillin–streptomycin were purchased from Thermo Fisher Scientific, Inc. (Wilmington, DE, USA). The primary antibodies for β-actin (ab8226) and MUC1 (ab109185) were purchased from Abcam (Cambridge, UK). BALB/c nude mice (female, 5–6 weeks old) were bought from Beijing Vital River Animal Center (Beijing, China). The goat anti-rabbit secondary antibody for MUC1 (ZB-2301) and anti-mouse secondary antibody for β-actin (ZB-2305) were obtained from ZSGB-Bio (Beijing, China).

### Preparation and characterization Fe_3_O_4_ and Fe_3_O_4_@DOP NPs

The monodisperse, hydrophilic Fe_3_O_4_ NPs were synthesized according to a reported solvothermal reduction method [33] with some modifications. In brief, 0.6 g of FeCl_3_·6H_2_O was dispersed in 30 mL of ethylene glycol. Next, 2.7 g of sodium acetate and 0.75 g of polyethylene glycol 2000 (PEG2000) were added sequentially to above solution under stirring for 60 min at 60 °C. Then, the solution was sealed into a Teflon-lined stainless-steel autoclave and heated in an oven at 200 °C for 16 h. The obtained Fe_3_O_4_ NPs were separated with a magnet, washed with ethanol and then deionized water each for 3 times. Finally, the Fe_3_O_4_ NPs were dried in a vacuum at 60 °C. To prepare Fe_3_O_4_@DOP NPs, 20 mg of Fe_3_O_4_ NPs were dispersed in 20 mL Tris–HCl buffer (10 mM, pH = 8.5) and 0.8 mg dopamine hydrochloride was added and sonicated for 5 min at room temperature. Then, the solution was kept stirring for 12 h. The resulted product was separated by using the magnet and washed with deionized water for 3 times and finally stored at 4 °C for further characterization.

Transmission electron microscopy (TEM) images of Fe_3_O_4_ NPs and Fe_3_O_4_@DOP NPs were performed using JEM-2100F (Japan) microscope operated at 200 kV. The scanning electron microscopy (SEM) images were obtained with a Hitachi SU-8020 microscope (Japan) operated at 20 kV. Hydrodynamic diameter and zeta potential were determined using the Zetasizer Nano ZS (UK). UV–vis absorption spectra were acquired using an UV–vis-NIR spectrometer (Cary500 Scan, Varian, USA). The structure of the nanoparticles was compared using a VERTEX70 Fourier Transform Infrared (FT-IR) spectrometer (Bruker Optics, Germany). The magnetic susceptibility properties of the prepared NPs were measured using a magnetometer equipment (Quantum Design-MPMS-XL7, USA) at 2–300 K with a 7T magnet.

### Quenching efficiency measurement

H_1_-FAM (10 nM) and H_2_-FAM (10 nM) were annealed in 1 × PBS buffer using the PCR instrument (Applied Biosystems, Thermo Fisher Scientific, USA) to form a stable hairpin, respectively. Then the annealed H_1_-FAM and H_2_-FAM were mixed with Fe_3_O_4_@DOP NPs at the final concentrations of 0, 0.1, 0.2, 0.4, 0.6, 0.8 or 1.0 mg/mL, and rotated at 25 °C for 10 min. The fluorescence spectrum (ex. 480 nm) of each mixture was collected using the Spark™ Multimode Microplate Reader (Tecan, Männedorf, Switzerland).

### Loading capacity of Fe_3_O_4_@DOP NPs for H-FAM

H_1_-FAM (50 nM) and H_2_-FAM (50 nM) were annealed in 1 × PBS buffer. Then the annealed H_1_-FAM and H_2_-FAM were mixed with Fe_3_O_4_@DOP NPs at the final concentrations of 0, 0.1, 0.2, 0.4, 0.6, 0.8 or 1.0 mg/mL, and rotated at 25 °C for 10 min. The DNA (H_1_-FAM and H_2_-FAM) after separation of Fe_3_O_4_@DOP NPs by magnet was measured using the NanoDrop OneC (Thermo Fisher Scientific, USA).

### Gel electrophoresis analysis of HCR products

For the standard HCR amplification, H1-FAM and H_2_-FAM were firstly annealed in 1 × PBS buffer, respectively. The HCR reaction mixtures including 15 μL 1 × PBS buffer, containing 500 nM H_1_-FAM, 500 nM H_2_-FAM, 0.5 M NaCl and T-mimic (0 nM, 25 nM or 50 nM) were incubated at room temperature for 4 h. Then, above HCR products were mixed with 3 μL 6 × Loading Dye before loaded into 10% native polyacrylamide gel electrophoresis (PAGE) for electrophoresis and stained using GelRed for visualization. The gels were analysed using imaging system (iBright FL1000, Thermo Fisher Scientific, USA).

### Optimization of the HCR amplification time

Firstly, H_1_-FAM and H_2_-FAM were annealed in PBS buffer, respectively. The mixtures in PBS buffer with a total volume of 15 μL containing 500 nM H_1_-FAM, 500 nM H_2_-FAM, 0.5 M NaCl and 100 nMT-mimic were incubated at 25 °C for 2 h, 4 h, 8 h or 12 h. The HCR products were visualized by PAGE electrophoresis.

### Cell culture

PANC-1 cells and HepG-2 cells were cultured in Dulbecco's Modified Eagle Medium (DMEM) containing 10% FBS. Capan-1 cells and BxPC-3 cells were maintained in Iscove's Modified Dulbecco's Medium (IMDM) and Roswell Park Memorial Institute (RPMI)-1640 medium complement with 10% FBS, respectively. HPDE-C7 cells were cultured in Modified Eagle Medium (MEM) containing 10% FBS. All the cells were maintained in a humidified incubator at 37 °C and under a 5% CO_2_ atmosphere.

### Cell counting

The cells were seeded in the 96-well plate and cultured until their confluence reached 70–80%. The cells were washed gently for three times with PBS buffer, and then fixed with 4% paraformaldehyde solution at 4 °C for 10 min (cell treatment protocol). After being washed with PBS for three times, random aptamer (Apt-random) or Apt-Tri-FAM (MUC1 probe) with the final concentration of 5 μM was added and incubated at RT for 120 min. Then, the Fe_3_O_4_@DOP NPs with the final concentration of 5 mg/mL were mixed with annealed H_1_-FAM (500 nM), H_2_-FAM (500 nM), 0.5 M NaCl and rotated at 25 °C for 10 min. The mixture was added to different concentration cells (0, 50, 10^2^, 10^3^, 10^4^ and 10^5^ cells/mL) and kept another incubation for 4 h before separated using external magnet, the fluorescence spectra of each supernatant was recorded under 480 nm excitation.

### Cell imaging

Cell imaging is the same as the protocol cell counting, except washing cells with PBS instead of Fe_3_O_4_@DOP NPs. The imaging of the cells was performed using the scanning laser confocal microscope (CLSM, Nikon A1, Japan). The Pearson’s correlation coefficient indicating the degree of colocation was analysed by the software of the CLSM. For the flow cytometry measurement, the cells after above treatment were diluted to 300 μL with PBS buffer before being subjected to the flow cytometer (Accuri C6 BD, Inc., Ann Arbor, MI, USA). For each sample, 20,000 events were collected and the fluorescence signals were detected with 488 nm laser excitation.

### Tissue imaging

All animal experiments were conducted under the guidelines of the Animal Care and Use Committee of Changchun Institute of Applied Chemistry, CAS, China (permission No. 69). Animal tumor model was set up as following: Capan-1, PANC-1, BxPC-3 or HepG-2 cells (each 1 × 10^7^ cells) suspended in 75 μL culture medium and 75 μL BD Matrigel Basement Membrane Matrix was subcutaneously injected into the anterior axillary fossa. When the volume reached about 100 mm^3^, the tumors were collected and fixed with 4% paraformaldehyde before embedded with paraffin. The tumor tissues were cut into slices for the following immunohistochemical staining (IHC) with primary MUC1 antibody and HRP conjugated secondary antibodies or fluorescent staining with our detection strategy. As for our staining method, the slices of tumor were firstly incubated with 20 μL of annealed Apt-Tri (5.0 μM) at 25 °C for 2 h and washed with PBS for 3 times. Then, tumor tissues were incubated with 20 μL of H_1_-FAM (2.0 μM), H_2_-FAM (2.0 μM) complexed with Fe_3_O_4_@DOP NPs, and 0.5 M NaCl at 25 °C for 4 h and then washed with PBS for 3 times. The nuclear were stained by incubating with 20 μL 1 × Hoechst33342 at 25 °C for 1 h.

### Bicinchoninic acid protein assay

The tumor tissues were washed gently with cold PBS buffer for three times to remove the blood stains, and then cut into small pieces. Appropriate volume of Radio-Immunoprecipitation Assay (RIPA) lysis buffer was added and the tissues were homogenized at 4 °C using the Tissue Grinding Tube. Total proteins were obtained by centrifugation for 10 min at 10,000*g* and quantified by Bicinchoninic acid (BCA) protein assay kit (Beyotime Biotechnology, China). The absorbance at 562 nm was recorded using a spectrophotometer (Spark™ Multimode Microplate Reader, Tecan, Switzerland).

### Western blotting

Each protein lysate was mixed with protein loading buffer and denatured in boiling water bath for 10 min. Then, each 30 μg protein was loaded into a sodium dodecyl sulfate–polyacrylamide gel electrophoresis (SDS-PAGE) containing the 5% stacking gel and 12.5% resolving gel, at 120 V for 2 h before being transferred to the 0.2 μm polyvinylidene fluoride (PVDF) membrane for hybridization (Millipore Corporation, USA). The membrane was blocked with 5% non-fat milk at room temperature for 3 h and then incubated the primary antibody of MUC1 or β-actin (1:1000 dilution) overnight at 4 °C. After incubating, the PVDF membrane was washed with TBST (20 mM Tris–HCl, 150 mM NaCl, 0.05% Tween 20) for 3 times and then incubated with HRP conjugated secondary antibodies (1:1000 dilution) at RT for 40 min. After washed with TBST, the protein bands were observed by chemiluminescent HRP substrates (ImmobilonTM Western, Millipore, USA) and then photographed using the gel performance system (iBright FL1000, Thermo Fisher Scientific, USA).

### Statistical analysis

All the experiments were performed at least 3 times independently and the data were presented as means ± SD and evaluated by one-way analysis of variance ANOVA using the software of GraphPad Prism 7.0 (USA).

## Results and discussions

### Working Principle for pancreatic cancer cell detection

**Scheme 1.**
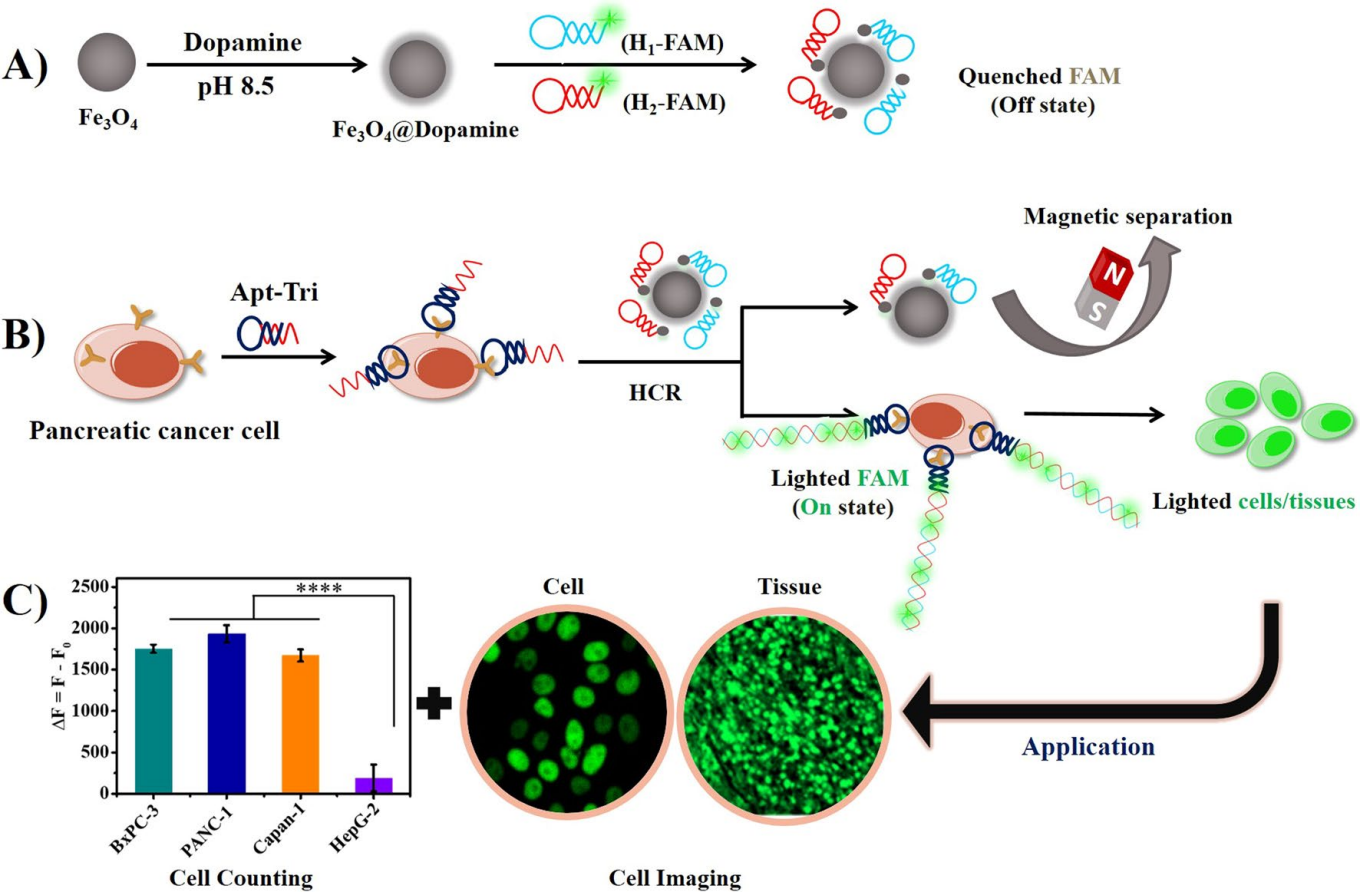
Schematic illustration for detection of pancreatic cancer cells. The preparation process of the Fe_3_O_4_@DOP NPs and the attachment of H_1_-FAM and H_2_-FAM onto the Fe_3_O_4_@DOP NPs (**A**). Diagram illustration for sensitive detection of pancreatic cancer cells based on Fe_3_O_4_@DOP NPs and HCR amplification (**B**). The application of the strategy in cell counting and imaging (**C**)

The proposed design is to apply HCR amplification with the help of MUC1 aptamer and Fe_3_O_4_@DOP NPs for specific recognition and detection of pancreatic cancer. As shown in Scheme 1A, Fe_3_O_4_ NPs are firstly coated with poly-dopamine at pH 8.5 in Tris–HCl buffer. Then, two FAM labelled hairpin DNA sequences (H_1_-FAM and H_2_-FAM) are added and the fluorescence of FAM is quenched by poly-dopamine after being absorbed on the Fe_3_O_4_@DOP NPs. Scheme 1B illustrates that in the presence of pancreatic cancers (high expression of MUC1 protein), the Apt-Tri probe, including two parts: a MUC1 aptamer (black segment) and a hybridization region, named trigger (red segment), will bind the MUC1 protein on the cells to form the cell/Apt-Tri complexes. With the addition of Fe_3_O_4_@DOP NPs absorbed by H_1_-FAM/H_2_-FAM, HCR reaction will be triggered on the cell surface. The H_1_-FAM and H_2_-FAM are pulled off from Fe_3_O_4_@DOP NPs, and the hairpin structures are opened through the hybridization between the trigger and H_1_-FAM/H_2_-FAM. As a result, a long and nicked duplex DNA product with lighted FAM is formed. Then, the extra quenched H_1_-FAM/H_2_-FAM DNA molecules attached on the Fe_3_O_4_@DOP NPs will be separated by external magnet.

After separation, HCR products with FAM molecules will stay on the pancreatic cancer cells for fluorescence measurement. As a result, the fluorescence intensity will be proportional to the concentration of pancreatic cancer cells. Therefore, the strategy can be used to quantify the concentration of pancreatic cancer cells in PBS buffer according to the calibration curve. Due to the strong fluorescent signal on cancer cells triggered by the HCR reaction, the designed scheme is also applicable to identify and image the MUC1-positive pancreatic cancer cells on tissues using CLSM (Scheme 1C). We thus conduct the tissue imaging directly in the pancreatic tumor to identify the MUC1-positive cells.

### Characterization of Fe_3_O_4_@DOP NPs

**Fig. 1:**
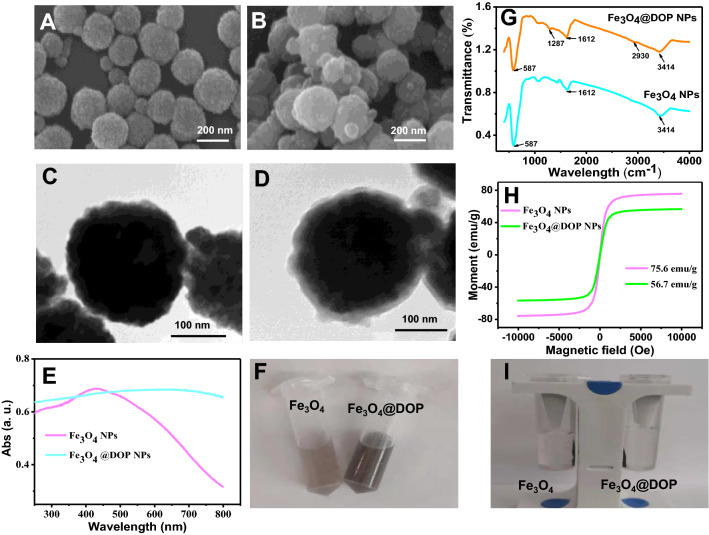
Characterization of the as-synthesized nanoparticles. SEM images (**A**, **B**); TEM images (**C**, **D**); UV–Vis absorption spectra (**E**), the optical photos (**F**), FT-IR spectra (**G**), magnetic hysteresis curves (**H**), and the magnetic properties measurement using the magnet (**I**) of Fe_3_O_4_ NPs and Fe_3_O_4_@DOP NPs

The as-prepared nanoparticles are characterized by SEM, TEM, UV–vis spectrometry, Fourier Transform infrared spectroscopy (FT-IR), and magnetic properties measurement system. As indicated in Fig. 1A and B, SEM images shows that both of Fe_3_O_4_ NPs and Fe_3_O_4_@DOP NPs are spherical with a nearly homogeneous size. The TEM images indicate a coating of a light contrast layer (6 nm) of poly-dopamine on the surface of Fe_3_O_4_ NPs (Fig. 1C and D). The mean sizes of Fe_3_O_4_ NPs and Fe_3_O_4_@DOP NPs measured by dynamic light scattering (DLS) are 220 ± 12.0 nm and 360 ± 48.6 nm, respectively (Additional file 1: Fig. S1A). The zeta potentials of Fe_3_O_4_ NPs and Fe_3_O_4_@DOP NPs were tested as (+ 16 ± 0.70) mV and (− 15 ± 0.71) mV, respectively (Additional file 1: Fig. S1B). The results of DLS and zeta potential measurements further prove the successful coating of dopamine on Fe_3_O_4_ NPs. The decrease of zeta potential after dopamine coating is resulted from the de-protonation of the phenolic hydroxyl and amino groups of the dopamine [34]. As shown in Fig. 1E, the absorption peak of Fe_3_O_4_ NPs is located at 430 nm and a broad featureless peak from 400 to 800 nm with high absorbance intensity is observed for Fe_3_O_4_@DOP NPs. Because dopamine hydrochloride has two free phenolic hydroxyl groups, which are easily oxidized to quinones under alkaline conditions, and it finally forms a black poly-dopamine layer on Fe_3_O_4_ NPs. After the dopamine being coated on the surface of Fe_3_O_4_ NPs, the colour of the solution becomes darker (Fig. 1F), which is consistent with the results of above UV–Vis spectra.

FT-IR spectra in Fig. 1G show that the bonds at 587 cm^−1^ and 3414 cm^−1^ are mainly related to the stretching vibrations of the Fe–O bond and the intra-molecular hydrogen bonding, respectively [35]. The peaks at the 1287 cm^−1^ and 2930 cm^−1^ are due to the presence of –NH_2_ and C–H stretching vibrations in the dopamine, which further confirms that Fe_3_O_4_@DOP NPs have been successfully synthesized. Figure 1H shows the obtained magnetic hysteresis curves of superparamagnetic Fe_3_O_4_ NPs and Fe_3_O_4_@DOP NPs at RT with no remnant magnetization, and the saturation magnetization (Ms) values of which are 75.6 and 56.7 emu g^−1^, respectively. The decrease of saturation magnetization value of Fe_3_O_4_@DOP NPs indicates that the non-magnetic dopamine is coated onto the Fe_3_O_4_ NPs [36]. However, it is still enough for the fast magnetic separation within 2 min and the Fe_3_O_4_@DOP NPs disperse quickly when removing the external magnet (Fig. 1I).

### Fluorescence quenching measurement

**Fig. 2:**
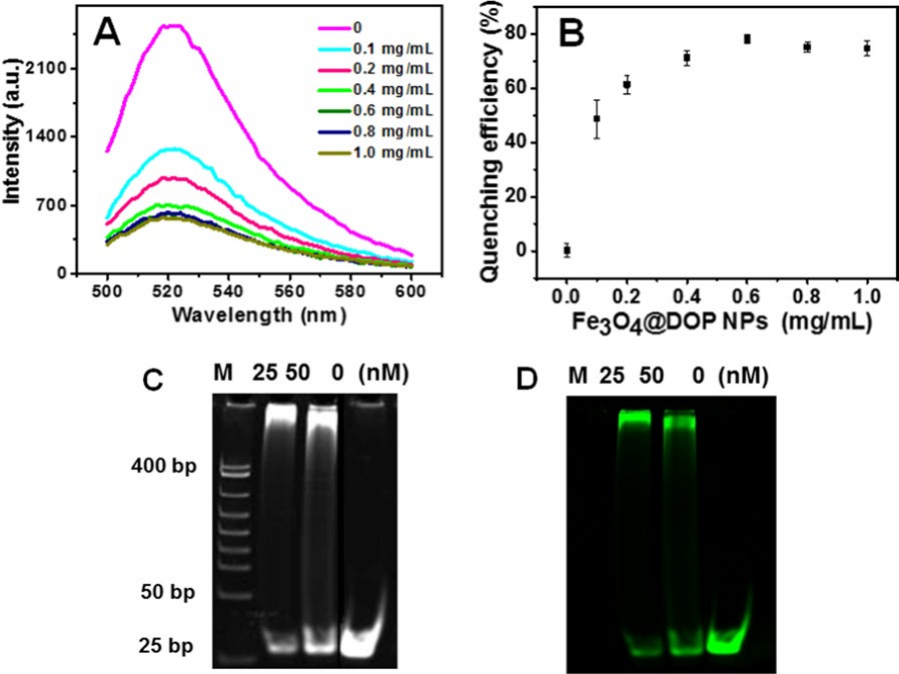
Fluorescence quenching ability measurement of Fe_3_O_4_@DOP NPs at different concentrations (0, 0.1, 0.2, 0.4, 0.6, 0.8, 1.0 mg/mL) on H_1_-FAM/H_2_-FAM before and after HCR amplification. **A**, **B** Quenching effect of Fe_3_O_4_@DOP NPs (0, 0.1, 0.2, 0.4, 0.6, 0.8 and 1.0 mg/mL) on the fluorescence of H_1_-FAM (10 nM) and H_2_-FAM (10 nM). The error bars represent the standard deviations (SD) of three independent experiments (n = 3). **C**, **D** HCR amplification evaluation using electrophoresis. The gel was stained with GelRed (**C**) or detected by the fluorescence of FAM (**D**). Lanes in both **C** and **D** showed the HCR product supplement with T-mimic at the concentrations of 25 nM, 50 nM, or 0 nM) containing 500 nM each H_1_-FAM and 500 nM H_2_-FAM. M: DNA marker

The fluorescence quenching ability of the Fe_3_O_4_@DOP NPs was firstly assessed using the mixture of H_1_-FAM (10 nM), H_2_-FAM (10 nM) and different concentrations of the Fe_3_O_4_@DOP NPs (Fig. 2A). Fe_3_O_4_@DOP NPs carrying H_1_-FAM and H_2_-FAM were separated by external magnet, and the fluorescent intensity of PBS solution is decreased accordingly. The percentage of fluorescence signal reduction was calculated using the equation: η = (1 − F/F_0_) × 100%, in which F and F_0_ are the fluorescence intensities (excitation: 494 nm) in the presence and absence of Fe_3_O_4_@DOP NPs, respectively. The results in Fig. 2B show that the fluorescence signal of FAM was reduced about 78.2% when the concentration of Fe_3_O_4_@DOP NPs reached to 0.6 mg/mL. This indicates the outstanding quenching ability of the Fe_3_O_4_@DOP NPs. The loading capacity of H-FAM to Fe_3_O_4_@DOP NPs was calculated to be 14.7 ng/mg Fe_3_O_4_@DOP NPs (Additional file 1: Fig. S2).

Since HCR can significantly improve the sensitivity of the proposed fluorescence sensor [37], we firstly applied T-mimic (the same sequence to the trigger) to verify the HCR process in our system. Then, we optimized the incubating time for HCR amplification to be 4 h (Additional file 1: Fig. S3). As shown in Fig. 2C and D, the HCR is initiated by the different concentrations of T-mimic. However, in the absence of T-mimic, H_1_-FAM and H_2_-FAM coexisted steadily with hairpin structure in solution without HCR product.

### Concentration determination of pancreatic cancer cells

**Fig. 3:**
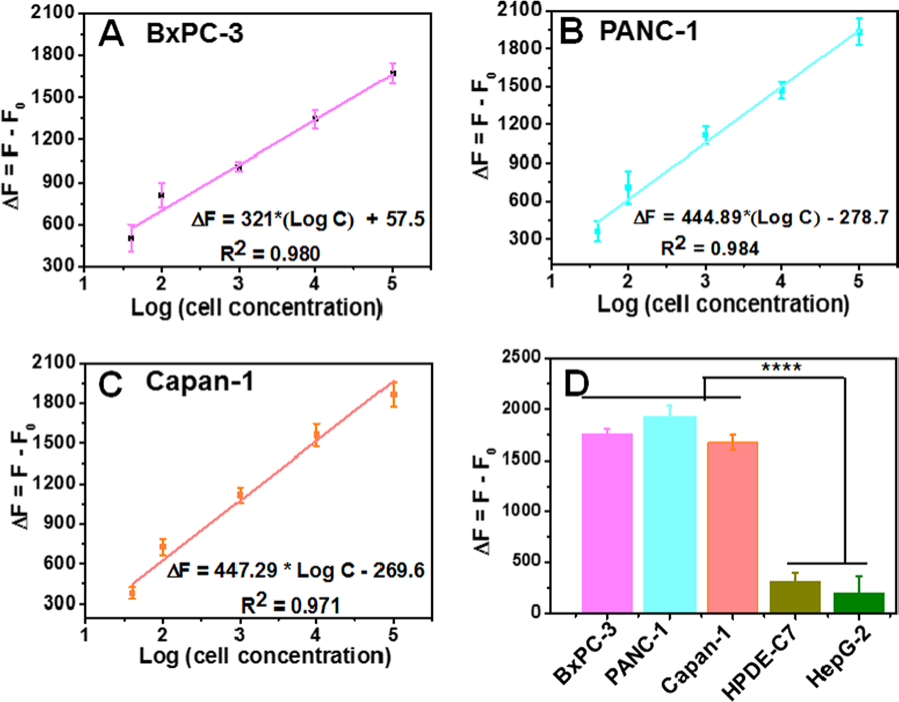
Sensitivity and specificity study. Calibration curves of fluorescence intensity versus the logarithm of different concentrations of pancreatic cancer cells (**A**–**C**). The fluorescence intensity comparison of the pancreatic cancer cells with HPDE-C7 and HepG-2 cells at the concentration of 10^5^ cells/mL (**D**). The error bars were obtained from three independent experiments (n = 3, ****P < *0.0001*)

The ability of this strategy to quantitatively detect the pancreatic cancer cells was also investigated. As shown in Additional file 1: Fig. S4A–C, the fluorescence intensity at 520 nm gradually increases with increasing concentration of pancreatic cancer cells (0–10^5^ cells/mL) except that of HepG-2 (Additional file 1: Fig. S4D) and HPDE-C7 cells (Additional file 1: Fig. S4E). Figure 3A exhibits a linear relationship between the fluorescence intensity and the concentration of BxPC-3 cells, and the regression equation is ΔF = 321 * Log C + 57.5 (ΔF = F − F_0_, F and F_0_ are the fluorescence intensities in the absence and presence of cells, respectively; Log C represents the logarithm of the cell concentration). The concentration ranges from 50 to 10^5^ cells/mL (R^2^ = 0.980) with the LOD of 21 cells/mL based on 3δ/S (δ is the standard deviation of the blank signal, S is the slope of the calibration curve) [38]. With the same detection strategy, the regression equations of PANC-1 (Fig. 3B) and Capan-1 cells (Fig. 3C) are ΔF = 444.89 * Log C− 278.7 (R^2^ = 0.984, LOD of 27 cells/mL) and ΔF = 447.29 * Log C − 269.58 (R^2^ = 0.971, LOD of 41 cells/mL), respectively. In general, compared with other reported pancreatic cancer cell detection methods [39, 40], the present work shows higher sensitivity and lower cost. The specificity of the assay was evaluated by the detection of four kinds of cell lines at the concentration of 10^5^ cells/mL. The fluorescence intensities for HepG-2 and HPDE-C7 cell are distinctly lower than that of the pancreatic cancer cell lines (Fig. 3D), indicating the good specificity of the proposed method.

### Pancreatic cancer cells imaging in vitro

As MUC1 protein highly express on pancreatic cancer cells [41], we decided to use our strategy in cell imaging. PANC-1 cells were chosen to optimize the incubating time of Apt-Tri-ROX. As indicated in Additional file 1: Fig. S5A, the highest intensity of ROX was obtained at 2 h, which was applied for the following investigation. As for the HCR, the relative highest fluorescence of FAM was observed at 4 h after the reaction, which indicates the best signal amplification time for cell imaging (Additional file 1: Fig. S5B).

**Fig. 4:**
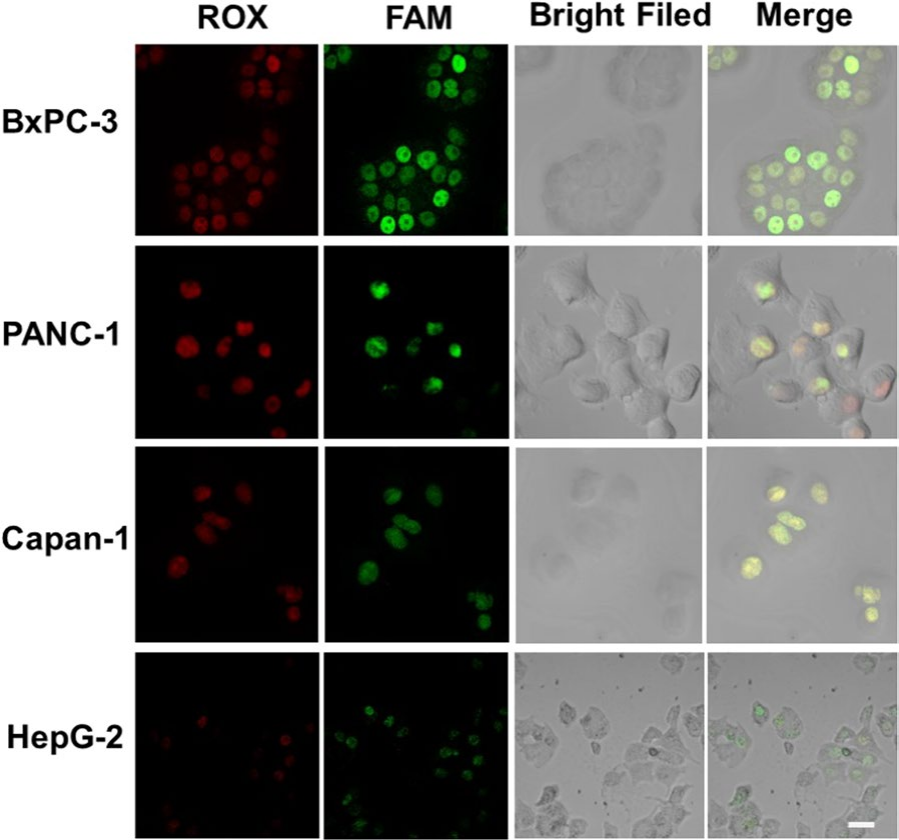
CLSM images of BxPC-3, PANC-1, Capan-1 and HepG-2 cells highlighted with Apt-Tri-ROX or Apt-Tri + H_1_-FAM/H_2_-FAM. Scale bar: 20 µm

Based on above conditions, the imaging of the three pancreatic cancer cell lines (BxPC-3, PANC-1 and Capan-1) and the HepG-2 cells (negative control) were explored (Fig. 4). MUC1 is a transmembrane glycoprotein overexpressed in the pancreatic cancer cells, and the subunits of MUC1 can be translocated into the nucleus to regulate the expression of other genes [42]. Apt-Tri-ROX could bind with MUC1 on the pancreatic cancer cells, leading to ROX fluorescence accumulation on the cells, and at the same time the Tri in the molecule probe was released for the HCR. Clearly, there was obvious red fluorescence in pancreatic cancer cells, after incubation with Apt-Tri-ROX, which was not conspicuous in the HepG-2 cells. This indicates the relative higher expression of MUC1 in pancreatic cancer cells than that in HepG-2 cells. The selectivity of our detection strategy was similar to the previous studies [43–45]. After further incubating with the HCR amplification solution, the Tri induced the HCR amplification, leading to enhanced FAM fluorescence signal, which can benefit the sensitivity of pancreatic cancer cell detection. Furthermore, the overlay images of the red and green channels also showed the co-localization of the MUC1 Apt and HCR products with the Pearson’s correlation coefficient of 0.86, 0.83, and 0.90 for BxPC-3, PANC-1 and Capan-1 cells, respectively, indicating that the HCR was initiated from and collocated with the Apt-Tri.

**Fig. 5:**
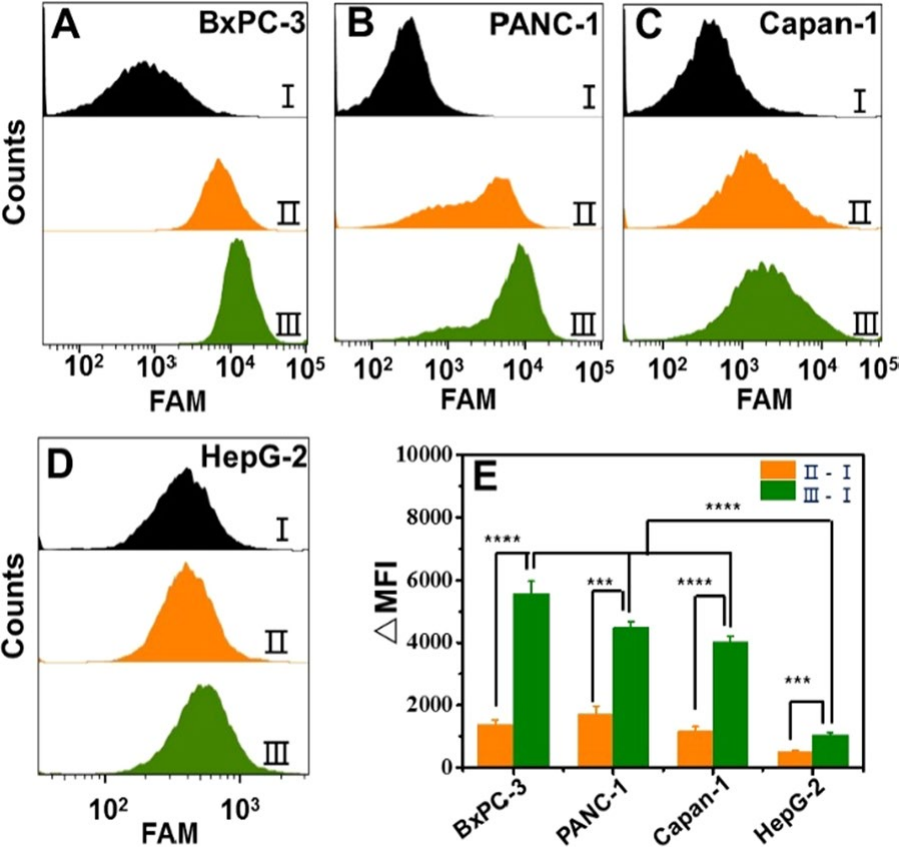
FCM analysis of cells incubated with no Apt (I), Apt-Tri-FAM (II) or further with HCR solution (III) for BxPC-3 (**A**), PANC-1 (**B**), Capan-1 (**C**) and HepG-2 (**D**) cells. The corresponding histogram showed the increase of the mean fluorescence intensity (MFI) for each pancreatic cell line before (II–I) and after HCR (III–I). (n = 3, ***P < *0.001*, ****P < *0.0001*)

To further confirm the signal amplification effect of HCR during the pancreatic cancer cells imaging, Apt-Tri-FAM instead of Apt-Tri-ROX was used for fluorescence intensity comparison using flow cytometry (FCM). As shown in Fig. 5, the enhancement of green fluorescence intensity of pancreatic cells after HCR (III-I) was significantly higher than that only treated with Apt-Tri-FAM (II-I). Moreover, the FCM results also showed that the mean fluorescence intensity (MFI) after HCR on the three pancreatic cancer cell lines was much higher than that on the HepG-2 cells (Fig. 5E). To further verify the specific of our strategy, we replaced Apt-Tri with Apt-random (a random DNA, completely different from the trigger sequence), and then amplified the signal with HCR in cell labelling. As shown in Additional file 1: Fig. S6, there is relatively low fluorescence in the cells after HCR, which indicates that the random DNA cannot trigger the HCR, although Apt-random is bound with the MUC1 protein by the Apt. These results also proved that the HCR amplification did boost the fluorescent intensity of the MUC1 positive pancreatic cells, which contribute to the early diagnosis of these types of tumors before their deterioration.

### Fluorescence imaging of pancreatic cancer tissue

**Fig. 6:**
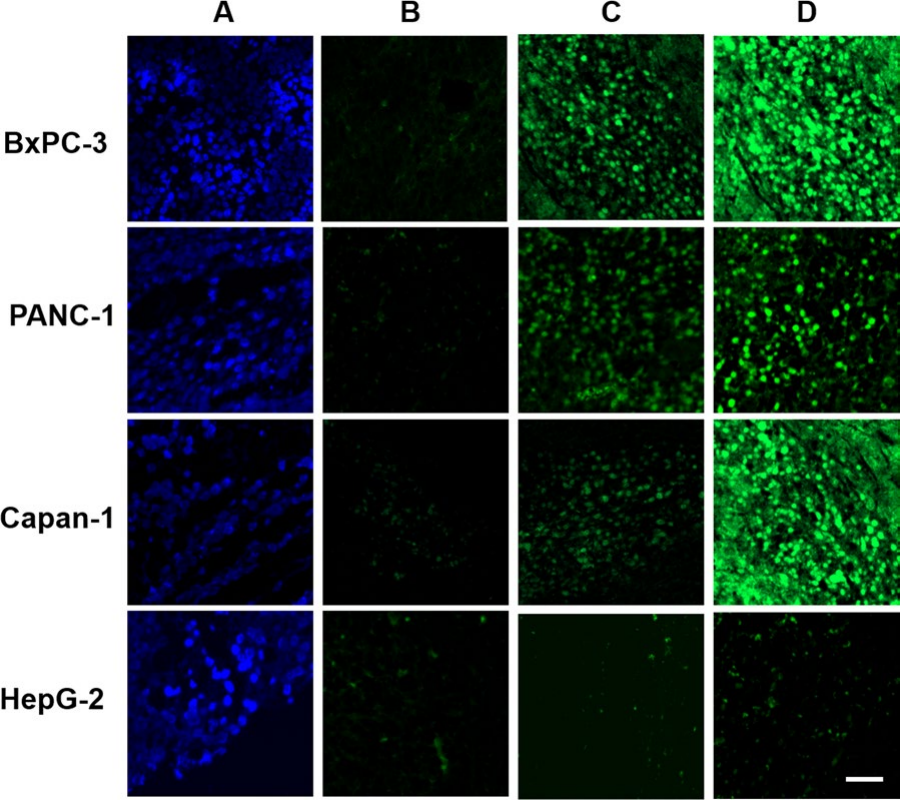
Fluorescence staining of tumor tissues implanted in nude mice by BxPC-3, PANC-1, Capan-1, or HepG-2 cells. Tissues were stained with Hoest33342 (**A**) and treated with Apt-random + H_1_-FAM/H_2_-FAM (**B**), Apt-Tri-FAM (**C**), or Apt-Tri-FAM + H_1_-FAM/H_2_-FAM (**D**), respectively. Scale bar = 20 µm

To verify the potential of clinical application of our system in pancreatic cancer diagnostic, we further performed the tissue staining in nude mice implanted by BxPC-3, PANC-1, Capan-1 and HepG-2 cells. As shown in Fig. 6, the green fluorescence in the cells treated with Apt-Tri + H_1_-FAM/H_2_-FAM (D) is much higher than those only incubated with Apt-random + H_1_-FAM/H_2_-FAM (B) or Apt-Tri-FAM (C) in BxPC-3, PANC-1 or Capan-1 cell-bearing tissues. While in the HepG-2 cell-bearing mice, the intensity of the green fluorescence is relatively lower than that in the pancreatic cancer cells, which is resulted from the less expression of MUC1 protein. These results are in accordance to the in vitro investigations shown in the cell imaging. Figure 6 also shows the similar results to those in Additional file 1: Fig. S4, and the pancreatic cancer cells has higher fluorescence intensity after HCR amplification in comparison that of with HepG-2 cells. The strategy can well distinguish the tissues derived from the pancreatic cancer cells out of that originated from HepG-2 cells.

To further confirm the results in tissue imaging, we performed the traditional western blot and immunohistochemical (IHC) staining, which indicate the expression of MUC1 on cancer cells-bearing tissues. As shown in Additional file 1: Fig. S7, the tissue derived from the pancreatic cancer cells has the higher expression of MUC1 compared with that coming from HepG-2 and HPDE-C7 cells. The results are consistent with the fluorescent imaging results shown in Fig. 6, which verifies that our method can be served as a new staining procedure for the identification of the pancreatic cancer cells on the tissues.

## Conclusions

In summary, we have demonstrated a new and easy operating strategy in sensitive and selective detection of MUC1 overexpressed pancreatic cancer. The strategy combines HCR with magnetic Fe_3_O_4_@DOP NPs to improve the detection sensitivity based on the signal amplification of HCR, quenching ability of dopamine layer and MUC1 Apt/cell recognition. The LODs reaches as low as 21–41 cells/mL of three pancreatic cancer cell lines. The high quenching ability of Fe_3_O_4_@DOP NPs plays a vital role in the deduction of fluorescence background on tissues. Since no enzyme is required in this strategy, the detection is low-cost and easy to operate. Moreover, the system is universal because it can be used in detection or imaging of MUC1 overexpressed cancer cells, and the presented detection approach can open new avenues on Apt based recognition of other pancreatic cancer biomarkers.

## Supplementary Information

**Additional file 1. **Additional figures and table.

## Data Availability

Not applicable.
