# Supplementary material for: Sensitive and selective detection of Mucin1 in pancreatic cancer using hybridization chain reaction with the assistance of Fe3O4@polydopamine nanocomposites
Source: J Nanobiotechnology. 2022 Feb 23;20:94. doi: 10.1186/s12951-022-01289-w (PMC8867748; doi:10.1186/s12951-022-01289-w)
Supplement: Supplementary file 1 — Additional file 1. Additional figures and table. [file 12951_2022_1289_MOESM1_ESM.docx]

**Supporting Information**

Sensitive and selective detection of Mucin1 in pancreatic cancer using hybridization chain reaction with the assistance of Fe_3_O_4_@polydopamine nanocomposites

Qing Dong,^1,2^ Xiuna Jia,^2^ Yuling Wang,^1^ Hao Wang,^1,2^ Qiong Liu,^2^ Dan Li,^2^* Jin Wang^4^* and Erkang Wang,^1,2^

^1^ College of Chemistry, Jilin University, Changchun 130012, Jilin, P. R. China

^2^ State Key Laboratory of Electroanalytical Chemistry, Changchun Institute of Applied Chemistry, Chinese Academy of Sciences, Changchun 130022, Jilin, P. R. China

^3^ ARC Centre of Excellence for Nanoscale BioPhotonics, Department of Molecular Sciences Macquarie University, Sydney, 2109, Australia

^4^ Department of Chemistry and Physics State University of New York at Stony Brook Stony Brook, NY 11794–3400, USA

* Corresponding author.

E-mail: [lidan@ciac.ac.cn](mailto:lidan@ciac.ac.cn); jin.wang.1@stonybrook.edu


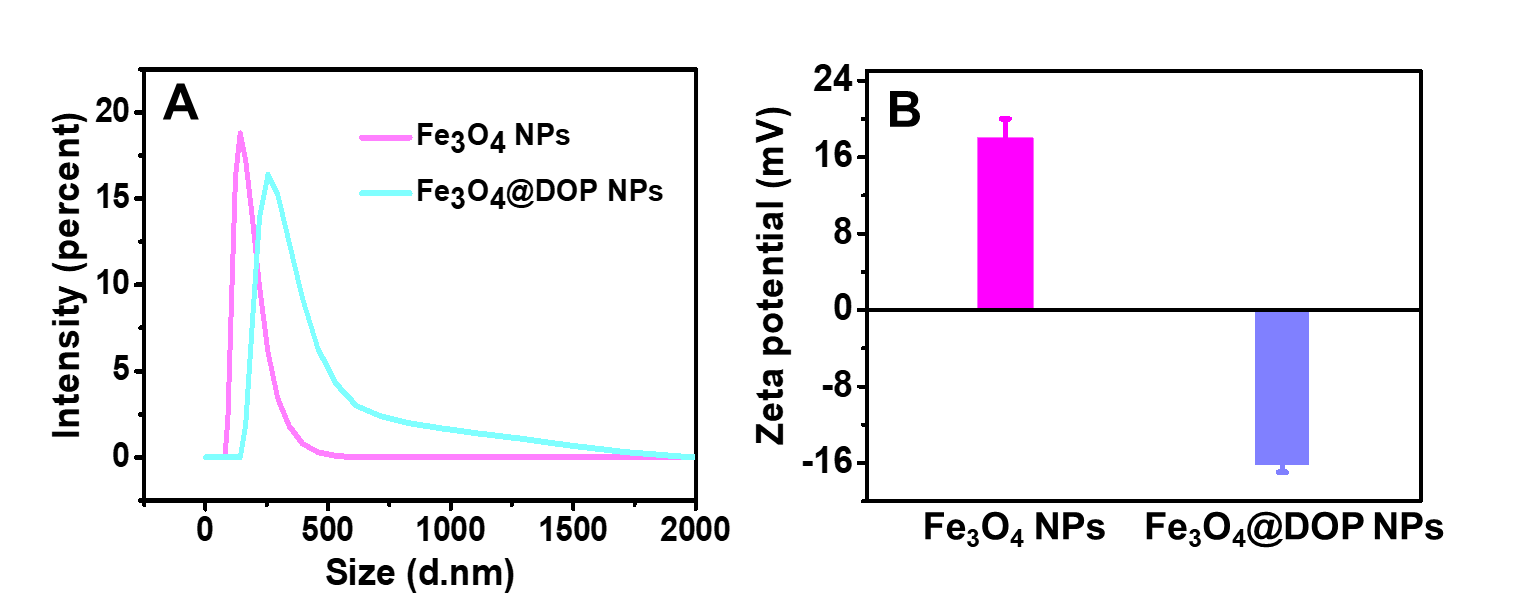


**Fig. S1.** DLS (A) and zeta potentials (B) of the Fe_3_O_4_ NPs and Fe_3_O_4_@DOP NPs.

**Fig. S2.** The loading capacity measurement of H-FAM for Fe3O4@DOP NPs.


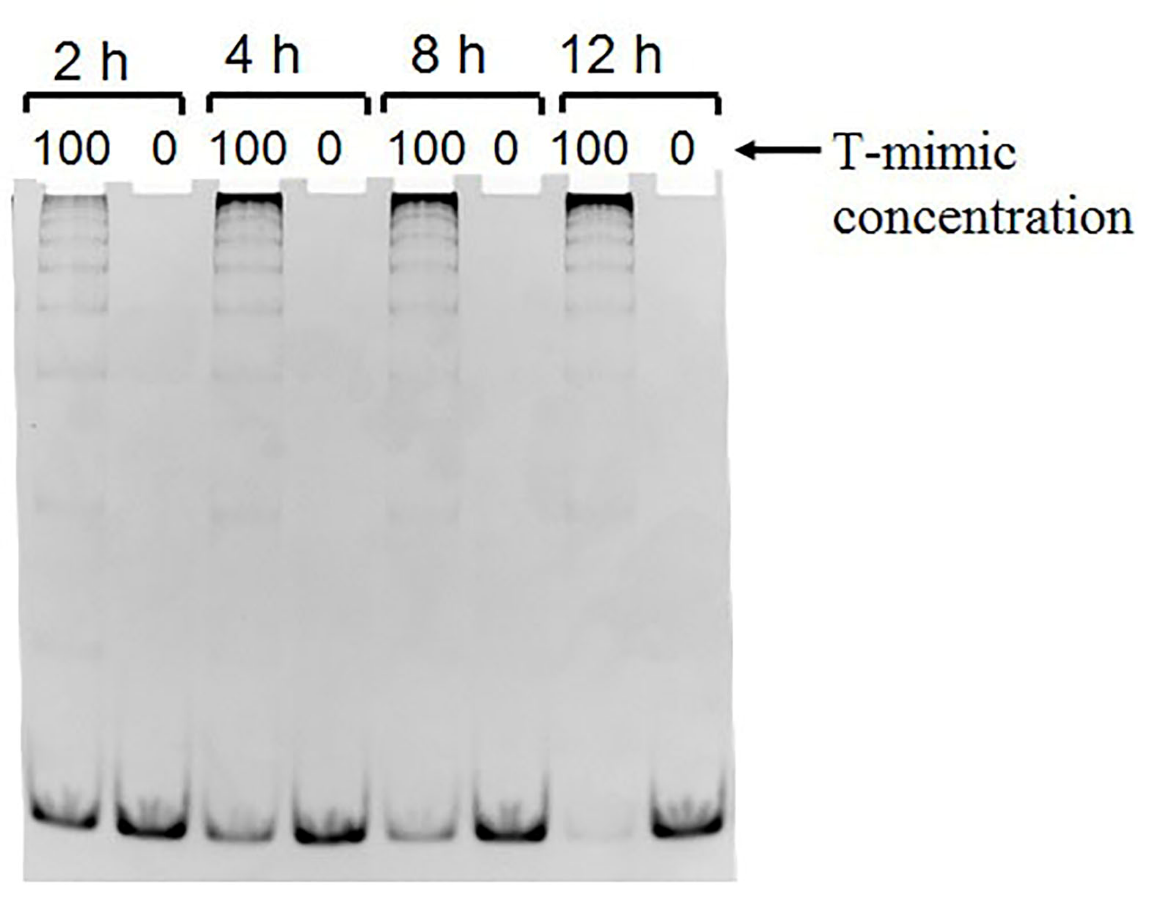


**Fig. S3.** The optimization of the incubating time of HCR amplification.


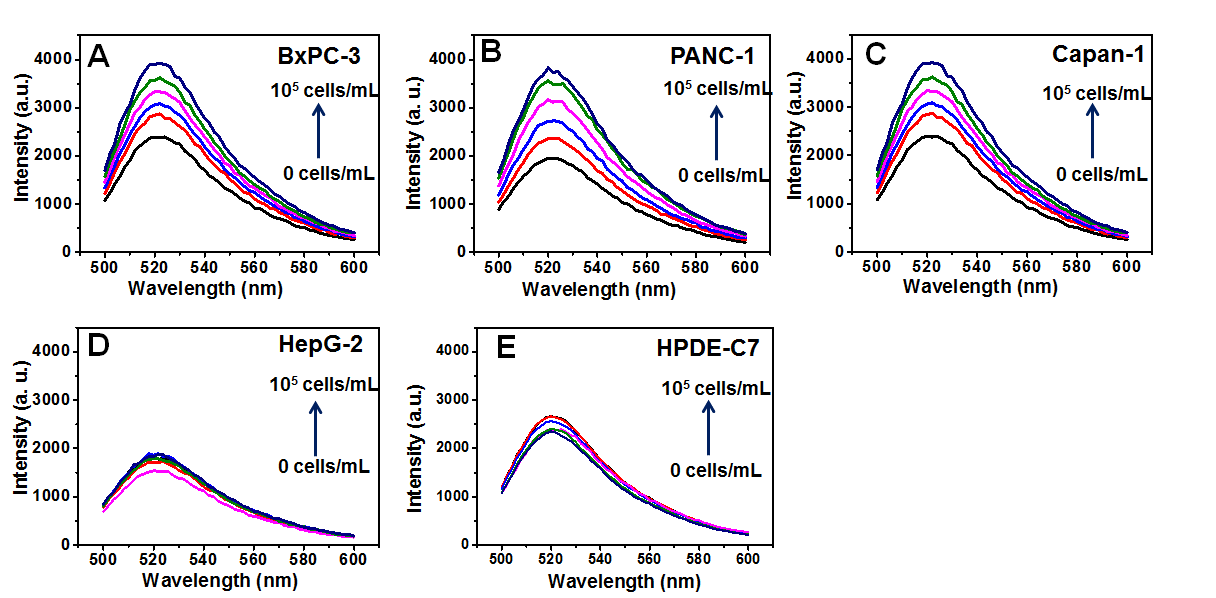


**Fig. S4.** Detection of pancreatic cancer cells (A, B and C), HepG-2 (D) and HPDE-C7 cells using fluorescence spectra, the concentrations of cells are 0, 50, 10^2^, 10^3^, 10^4^ and 10^5^ cells/mL.


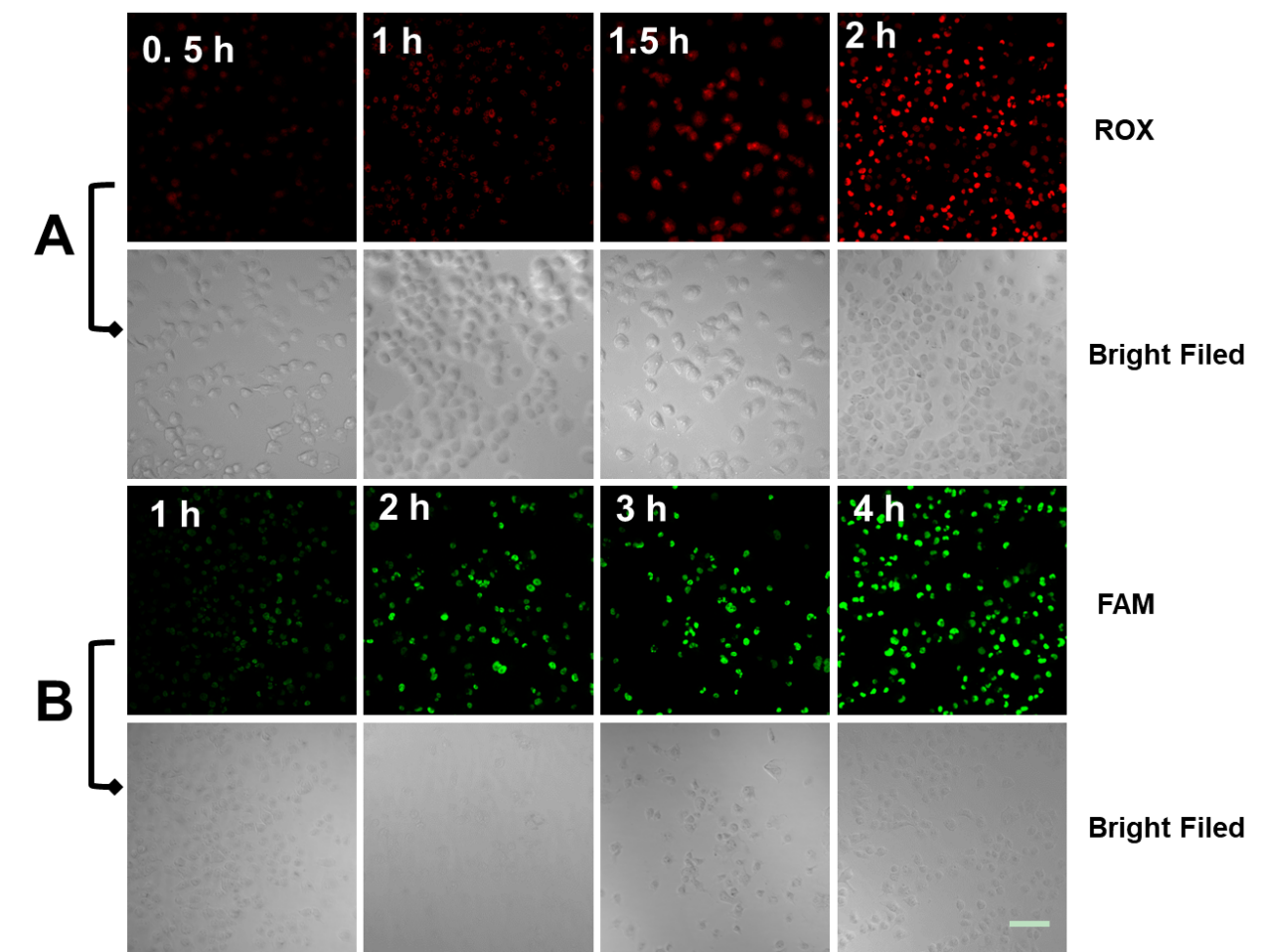


**Fig. S5.** Optimization of the time of Apt-Tri-ROX binding and HCR amplification on PANC-1 cell monitored by CLSM. (A) The concentration of Apt-Tri-ROX was 1.0 µM, and the incubation time was 0.5 h, 1 h, 1.5 h and 2 h. (B) HCR time was 1 h, 2 h, 3 h and 4 h, scale bar = 100 μm.


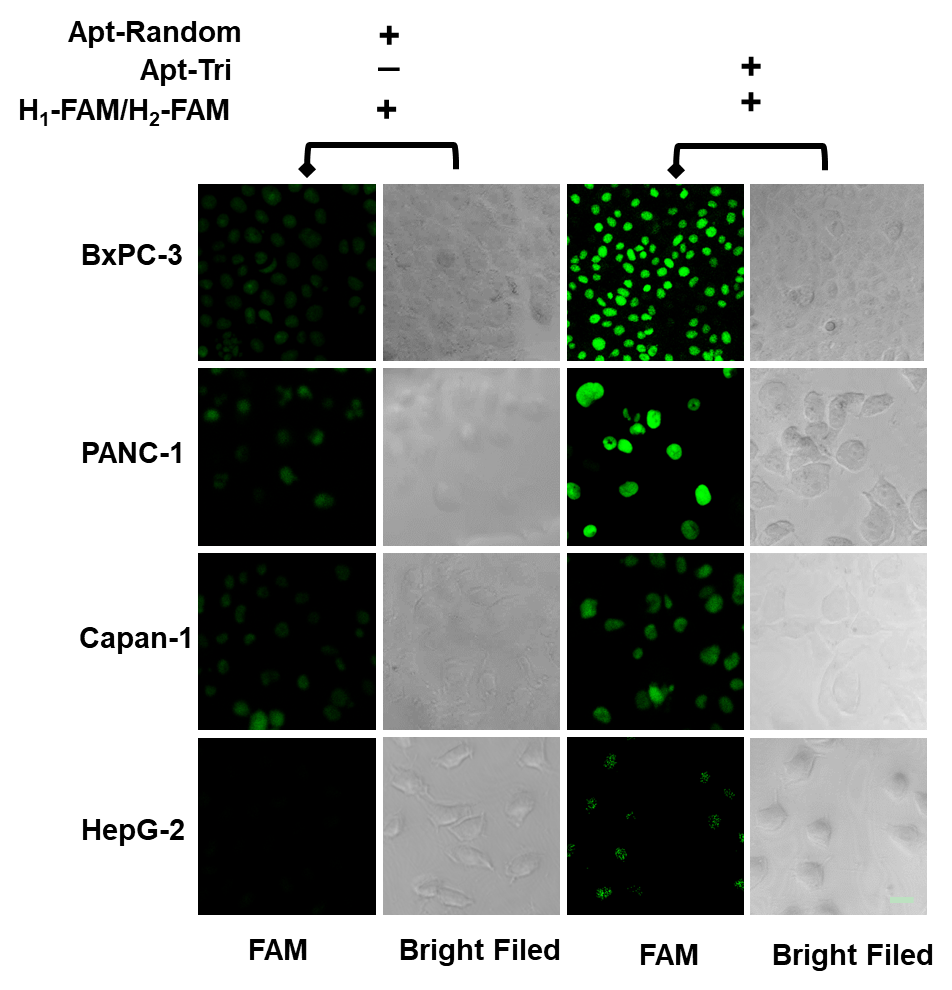


**Fig. S6.** CLSM images of BxPC-3, PANC-1, Capan-1 and HepG-2 cells treated with Apt-Random + H_1_-FAM/H_2_-FAM or Apt-Tri + H_1_-FAM/H_2_-FAM, scale bar = 20 μm.


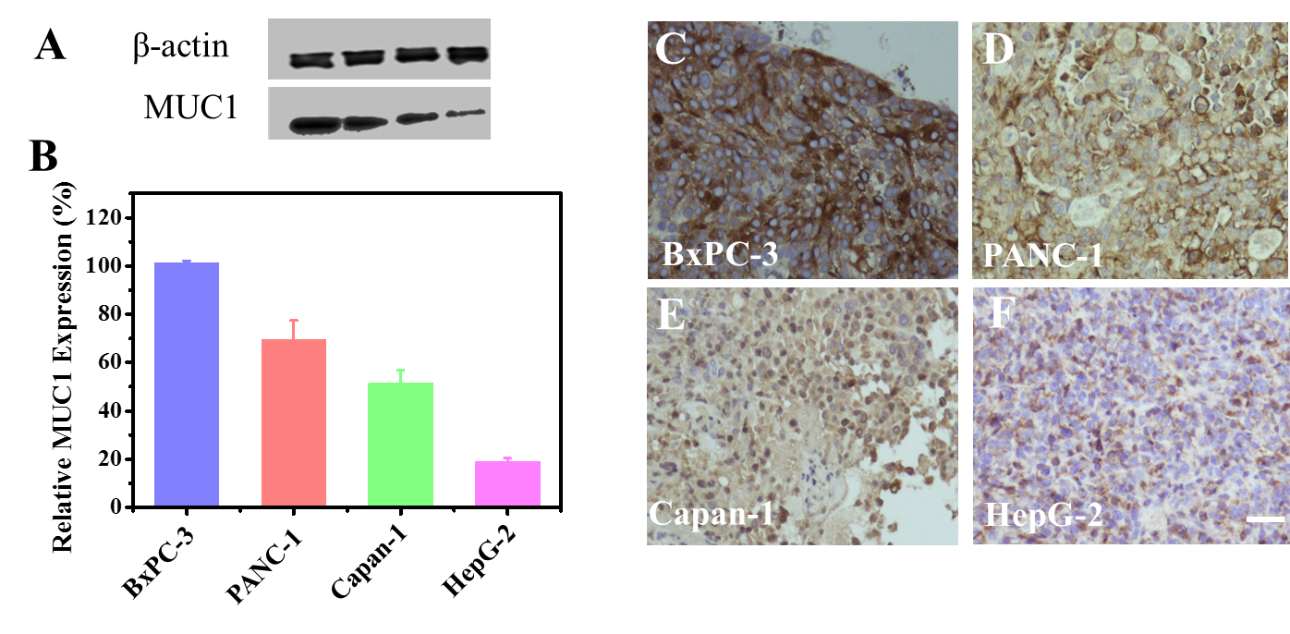


**Fig. S7**. Western blot (A, B) and IHC staining images show the expression of MUC1 in the tumor tissues derived from BxPC-3 (C), PANC-1 (D), Capan-1 (E), and HepG-2 cells (F). Scale bar = 100 µm.

**Table S1. Oligonucleotides used in this paper**

| Name | Sequences (5’- 3’) |
| --- | --- |
| Apt-Tri-FAM | /6-FAM-GCAGTTGATCCTTTGGATACCCTGGTTTTTTTTTT  TTTGATCAACTGCTAGCTTATCTG |
| Apt-Tri-ROX | /6-ROX-GCAGTTGATCCTTTGGATACCCTGGTTTTTTTTT  TTTTGATCAACTGCTAGCTTATCTG |
| H_1_-FAM | /6-FAM-CAGATAAGCTAGCAGTTGATCAGATTATTGATCAA  CTGCTAGCT |
| H_2_-FAM | /6-FAM-TGATCAACTGCTAGCTTATCTGAGCTAGCAGTTG  ATCAATAATC |
| Trigger  (T-Mimic) | GATCAACTGCTAGCTTATCTG |
| Apt-random-ROX | /6-ROX-ATAAGGAACGTGCTGCTACTCATCCTAGTCGAGA  CCACAACGGTTTCCCTG |
